# Role of Exopolysaccharides of *Pseudomonas* in Heavy Metal Removal and Other Remediation Strategies

**DOI:** 10.3390/polym14204253

**Published:** 2022-10-11

**Authors:** Katarína Balíková, Hana Vojtková, Eva Duborská, Hyunjung Kim, Peter Matúš, Martin Urík

**Affiliations:** 1 Institute of Laboratory Research on Geomaterials, Faculty of Natural Sciences, Comenius University in Bratislava, Ilkovičova 6, 84215 Bratislava, Slovakia; 2 Department of Environmental Engineering, Faculty of Mining and Geology, VŠB–Technical University of Ostrava, 17. Listopadu 15/2172, 70800 Ostrava, Czech Republic; 3 Department of Earth Resources and Environmental Engineering, Hanyang University, 222 Wangsimni-ro, Seongdong-gu, Seoul 04763, Korea

**Keywords:** exopolysaccharides, *Pseudomonas*, biosorption, bioremediation, heavy metals

## Abstract

*Pseudomonas* biofilms have been studied intensively for several decades and research outcomes have been successfully implemented in various medical and agricultural applications. Research on biofilm synthesis and composition has also overlapped with the objectives of environmental sciences, since biofilm components show exceptional physicochemical properties applicable to remediation techniques. Especially, exopolysaccharides (ExPs) have been at the center of scientific interest, indicating their potential in solving the environmental issues of heavy metal land and water contamination via sorptive interactions and flocculation. Since exposure to heavy metal via contaminated water or soil poses an imminent risk to the environment and human health, ExPs provide an interesting and viable solution to this issue, alongside other effective and green remedial techniques (e.g., phytostabilization, implementation of biosolids, and biosorption using agricultural wastes) aiming to restore contaminated sites to their natural, pollution-free state, or to ameliorate the negative impact of heavy metals on the environment. Thus, we discuss the plausible role and performance of *Pseudomonas* ExPs in remediation techniques, aiming to provide the relevant available and comprehensive information on ExPs’ biosynthesis and their usage in heavy metal remediation or other environmental applications, such as wastewater treatment via bioflocculation and soil remediation.

## 1. Introduction

Bacterial exopolysaccharides (ExPs), a group of extracellular polymeric substances (EPSs), are the structural and functional components of microbial biofilms that display exceptional physicochemical properties. Thus, bacterial ExPs with unique attributes have found their way into biomedical science practice (e.g., tissue engineering) and have been successfully implemented into a myriad of industrial and medical applications [1,2].

Interest in ExP-producing bacteria has also expanded into the research areas of environmental sciences, including studies on eco-friendly municipal and wastewater treatment processes [3]. This is because many standard remediation techniques require the usage of reagents that may be hard to degrade, or various environmentally harmful by-products are produced during their utilization at contaminated sites. This includes chemical treatment, which usually detoxifies metals via redox transformation or neutralization by application of the reagents, such as potassium permanganate, hydrogen peroxide, hypochlorite, synthetic surfactants, or chlorine gas, to precipitate, immobilize, or preconcentrate the hazardous contaminants [4,5,6]. The chemical leaching of soils and sediments by applying strong inorganic and organic acids and persistent synthetic chelating agents (e.g., ethylenediaminetetraacetic acid or its derivatives) to solubilize contaminants has also been successfully tested for heavy metal removal [7]. Reactive solid inorganic and biological substances, as well as materials with active surfaces (e.g., zero-valent iron, ferric oxides and oxohydroxides, nanomaterials, zeolite, biological waste) have been studied as potential components of permeable treatment barriers to restrict the movement of the contaminant in the environment [8,9,10,11]. However, in some cases, unpredictable effects regarding the toxicity and mobility of generated species can be expected since these interactions are usually non-specific. The application of electrochemical and electrokinetic remediation methods, engineered to site-specific requirements, has been performed for heavy metal removal [12], showing promising results in combination with other remediation approaches, including novel biochemical methods [13]. More prominent green approaches include phytoremediation, phytoextraction, and biosorption. They are usually performed in conjunction with other methods, e.g., chemical leaching [14]. Still, the application of microbial ExPs is environmentally advantageous since these biogenic polymers are usually water soluble, susceptible to natural degradation, and less harmful than synthetic polymers [15].

Bacterial ExPs find their successful applications in heavy metal removal, oil recovery, and various in situ remediation techniques such as emulsifiers, sorbents, biofilters, surfactants, and bioflocculants [16,17,18]. The interest of environmental researchers in ExP-producing bacteria is also highlighted in several patent deposits focusing on the prosperous application of bacteria in the remediation of contaminated sites. Villela et al. [19] reported that there are 114 patents describing the degradation of oil compounds exclusively by *Pseudomonas*, thus highlighting the leading role of this bacterial genus in hydrocarbon-contaminated site remediation.

The utilization of *Pseudomonas* strains in remediation is not limited solely to the biodegradation of hydrocarbons; they have also been successfully applied for the decontamination of heavy metal-polluted waters, soils, and sediments [20]. This is primarily due to their ability to produce metal-chelating siderophores and surface-active extracellular polymeric substances [21]. Regarding the latter, *Pseudomonas* species are considered high-ExP-producing organisms [22], and since they are ubiquitous, being isolated from various types of environments, including industrial waste and activated sludge [23], they are considered potent in solving the issue of heavy metal contamination [24]. Unfortunately, to our knowledge, no exhaustive review has been published that specifically explores the interaction of heavy metals with ExPs of *Pseudomonas*. Therefore, this review aims to sum up this topic by providing comprehensive information on ExPs’ biosynthesis and the usage of these Gram-negative, aerobic bacilli in heavy metal remediation as well as some other environmental applications (e.g., soil stabilization and turbidity decrease).

## 2. Biosynthesis of Extracellular Polysaccharides in *Pseudomonas*

**Figure 1:**
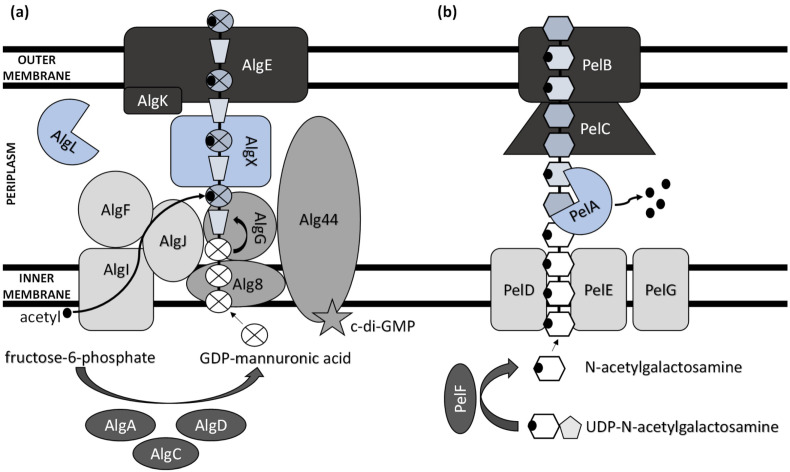
Schematic model of (**a**) alginate and (**b**) Pel polysaccharide biosynthetic machinery.

Extensive progress has been made in elucidating the synthesis of bacterial extracellular homopolysaccharides and heteropolysaccharides in recent years (Figure 1). They are synthesized by bacteria either extracellularly (outside the cell membrane and the cell wall), within the cell wall, or intracellularly [25].

While heteropolysaccharides are mostly synthesized intracellularly and transported outside the cell, homopolysaccharide production generally involves the activity of enzymes secreted by the bacterium to the extracellular environment. ExP production comprises several steps, including the synthesis of ExP precursors, repeat-unit assembly on a lipid carrier located at the cytoplasmic membrane, modification (e.g., acylation, acetylation, sulphation, and methylation), membrane translocation, polymerization, and export [26]. Thus, there are several functionally distinguished enzymes required for ExPs’ synthesis and development [27]. Several other enzymes, which are not unique to ExP production and serve as intermediates in protein regulation and central carbon metabolism, are also involved in ExPs’ biosynthesis process [28].

The biosynthesis of ExPs requires the involvement of activated monosaccharides derived from catabolized sugars. These include sugar nucleosides (nucleoside diphosphate sugars) or their derivatives (e.g., uridine diphosphate (UDP)-N-acetylglucosamine and guanosine diphosphate (GDP)-mannuronic acid) [29].

The main ExP component in the bacteria of *Pseudomonas* genera is alginate, an anionic linear polymer composed of beta-1,4-linked mannuronic acids (M-blocks) and C5-epimer α-L-guluronic acid (G-blocks) [30]. Alginate’s viscosifying, gelling, and stabilizing properties make this biopolymer an important industrial polysaccharide with great prospects in applications such as drug and protein delivery systems and food encapsulation [31,32]. Thirteen proteins are directly involved in the biosynthesis of alginate, and except for AlgC, they are all encoded by the *alg* operon [33]. The alginate precursor is synthesized by three enzymes including AlgA (bifunctional enzyme phosphomannose isomerase/guanosine 5′-diphospho-D-mannose pyrophosphorylase), AlgC (phosphomannomutase) and AlgD (GDP-mannose dehydrogenase), which allow the conversion of fructose-6-phosphate to GDP-mannuronic acid via four steps [34,35,36]. Alginate is first synthesized as a linear homopolymer from the GDP-mannuronic acid to polymannuronic acid by catalytic subunit Alg8 (alpha-1,3-glucosyltransferase) which interacts with Alg44 co-polymerase located at the cytoplasmatic membrane [37], with most of the latter being exposed to the periplasm [38]. These enzymes allowed the movement of the alginate precursor across the inner membrane for polymerization [39]. Alg44 also demonstrated the capability of binding the second messenger cyclic dimeric guanosine monophosphate (c-di-GMP) synthesized by MucR, a membrane-anchored protein, which is required for alginate biosynthesis [40].

In the periplasm, the polymannuronate is modified by epimerization or acetylation. AlgI, AlgJ, and AlgF are required for the addition of O-acetyl groups to the alginate polymer at O2 and/or O3 positions, which is an essential process for the stabilization of the intracellular alginate matrix for microcolony formation [41]. Acetylation can also affect epimerization reactions, since the activity of AlgG, a C5-epimerase that directly converts D-mannuronate to L-guluronate, has been detected only when the acetyl groups are removed from the polymannuronate substrate [42]. Newly formed macromolecules are most likely transported within the periplasm by the periplasmic protein AlgX that surrounds and protects the polymers from degradation by AlgL, a periplasmic alginate lyase [43,44]. Alginate is then secreted by the putative export protein AlgE [45]. The proper localization of AlgE for the periplasmic components of the alginate’s biosynthetic machinery is facilitated by AlgK [46].

The acid hydrolysis method is usually applied for the determination of ExPs’ monomeric components (e.g., glucose, fructose, galactose, and arabinose). Myszka and Czaczyk [47] reported that, under starvation conditions (ABPG medium reduced by 90% (*w*/*v*) of optimal nutrient availability), the EPS matrix of *Pseudomonas aeruginosa* ATCC 10145 consisted solely of glucosyl units. Grob, et al. [48] suggested that the survival of *P. aeruginosa* SG41 under highly chlorinated conditions was enabled by the overproduction of alginate, a major component of the SG41 strain’s ExPs (109.8 µg·g^−1^ cell dry mass) [49]. Thus, alginate overproduction is advantageous in harsh environments. Still, the nonmucoid *P. aeruginosa* strains that are the predominant environmental phenotype do not need to express the alginate biosynthetic genes to form the nonmucoid biofilms [50]. These use either Pel or Psl as the primary matrix structural polysaccharide [51].

A previously reported analysis of Pel polymer suggested that it is rich in cationic amino sugars, N-acetylgalactosamine, and N-acetylglucosamine, in a 5:1 ratio [52]. However, just recently, Le Mauff et al. [53] characterized the configuration and structure of Pel and suggested that it is a polymer of partially de-N-acetylated α-1,4-N-acetylgalactosamine, and it does not contain N-acetylglucosamine.

Pel synthesis requires protein products of a seven-gene operon *pelABCDEFG* [54]. The protein complex of PelD, PelE, PelF, and PelG is very likely a Pel-polysaccharide synthase whose activity is dependent on the localization of cytosolic glycosyltransferase PelF [55] to the inner membrane protein complex PelDEG [56]. PelDEG is also competent for the transport of the Pel polymer across the cytoplasmatic membrane.

PelA is a multi-domain protein that localizes to both the periplasm and membrane and exhibits both hydrolase and de-N-acetylase activity [57,58]. The PelBC complex is responsible for the transport of the matured polymer into the extracellular milieu. PelB is located at the outer membrane and contains a transmembrane β-barrel porin towards which the lipoprotein PelC, which is localized to the inner leaflet of the outer membrane, guides the positively charged Pel and, thus, acts as a charged molecular funnel facilitating Pel export [59].

Psl is a neutral branched pentasaccharide comprising D-mannose, D-glucose, and L-rhamnose. It is synthesized by the *p*olysaccharide *s*ynthesis *l*ocus (*psl*). The *psl* gene cluster consists of 15 genes, of which 11 are necessary for Psl polysaccharide synthesis (*pslACDEFGHIJKL*) [60]. However, the specific function of each protein is not completely understood.

*pslB* likely encodes a bifunctional enzyme with GDP-mannose pyrophosphorylase/phosphomannose isomerase dual activities, which is the only enzyme from the *psl* operon involved in sugar nucleotide precursor production [61]. The inner membrane-associated glycoside hydrolase PslG can be involved in the biosynthesis of Psl polysaccharide [60], although its role in this process is controversial [62]. Since the five PslAEJKL proteins have inner membrane-spanning domains, it was hypothesized that they make up the Psl polymerization complex [33]. Regarding Psl translocation and export, the complex of PslD and PslE helps transport Psl across the outer membrane [63].

## 3. Environmental Applications of ExPs

### 3.1. Heavy Metal Removal

The heavy metal removal from various environmental matrices by microbial biomass has been studied extensively in recent years. In the case of *Pseudomonas* strains, the application of bacterial extracellular polymeric substances, including purified ExPs, is at the center of interest as an alternative approach for the remediation of contaminated waters, since these extracellular biomolecules are capable of adsorbing and flocculating various metal ions effectively and with enormous environmental and economic advantages, as discussed in the following text.

Lau et al. [64] partially purified the capsular ExPs of *Pseudomonas* sp. CU-1 and monitored the copper(II) binding onto the cells via the dye displacement method. They concluded that the ExPs efficiently prevented the contact of copper with the cell surfaces since the 0.32 mmol·g^−1^ sorption capacity for copper(II) was only slightly lower than the removal capacity of the cells’ pellets (0.33 mmol·g^−1^). This is in good agreement with Kazy et al. [65], who noted that the production of ExPs was more pronounced in the copper-resistant *Pseudomonas aeruginosa* strain (4.78 mg mg^−1^ cell dry wt.) in comparison with the copper-sensitive strain (2.78 mg mg^−1^ dry wt.). Furthermore, the ExPs of the resistant strain could accumulate 1.2-fold higher amounts of copper(II) in comparison with its copper-sensitive counterpart.

The sequestration of copper(II) in EPSs efficiently prevents its access to the cytoplasm since the cell fractionation revealed that the cytoplasmic copper(II) was significantly higher in sensitive cells of *P. aeruginosa* [66]. Therefore, the optimization of ExP production by *P. aeruginosa* is a reasonable strategy for the sequestration of heavy metals. Chug et al. [67] reported that up to 26 mg (dry wt.) of *P. aeruginosa* strains’ ExPs can be obtained after 96 h incubation at pH 6 and 32 °C temperature in 50 mL of culture media. It was also tested against nickel(II) and chromium(VI); however, the outcomes were not satisfactory since only 26% and 9% of chromium(VI) and nickel(II) were removed from the aqueous systems at pH 7 when the initial concentration of metals in the solution was 10 mg·L^−1^.

The ExPs with high flocculating activity produced during submerged fermentation with high metal resistance bacterium *P. aeruginosa* Al-Dhabi144 [68] have been exploited for the removal of heavy metals from industrial wastewater. They exhibited various levels of metal removal efficiencies since the metal-binding activities varied with the type of metal being used in this study, including 1 mM concentrations of copper(II), cadmium(II), lead(II), cobalt(II), and zinc(II). The sorption capacities of ExPs for lead, copper, cadmium, zinc, and cobalt cations reached 380 mg·g^−1^, 300 mg·g^−1^, 250 mg·g^−1^, 250 mg·g^−1^, and 225 mg·g^−1^ of ExPs, respectively. Interestingly, Kumari and Das [69] observed that the densities of biofilm-associated polysaccharide components were reduced considerably in the EPS of *P. aeruginosa* N6P6 after being treated with lead(II). This ultimately reduced the overall biofilm density. This is especially important for heavy metal removal since high-volumetric-density biofilms generally exhibit higher sorption capacities in comparison with low-density biofilms that are characterized by the low content of ExPs [70]. Similarly, Abinaya Sindu and Gautam [71] noted that the exposure of the *P. aeruginosa* MTCC 2297 strain to metal fatty acid salts that included cadmium(II) stearate led to poor development of the biofilm with the least amount of ExPs. Therefore, only 1.1% of cadmium(II) was absorbed by the biofilm ExPs, while almost 58%, 52%, and 48.5% of zinc(II), copper(II), and iron(III) were removed by the ExPs, respectively. The complexation of metal ions with the carboxyl and phosphate functional groups of ExPs using FTIR analysis has been revealed.

The critical role of ExPs in metal sequestration was also highlighted by Rizvi and Saghir Khan [72], who suggested that these biomolecules maintain the metabolic activity of Gram-negative *P. aeruginosa* CPSB1 (and *Azotobacter chroococcum* CAZ3) even under stressful conditions of heavy metal contamination, and prevent desiccation as well. Thus, ExP production by *P. aeruginosa* CPSB1 increased by at least 34% when it was exposed to 200 µg·mL^−1^ of copper(II), cadmium(II), chromium(VI), nickel(II), or lead(II), relative to the untreated control.

Ferreira et al. [73] noted that the soluble ExP fraction of EPSs produced by *Pseudomonas veronii* 2E was composed of fucose, galactosamine, glucosamine, galactose, glucose, mannose, and glucuronic acid. It was capable of removing 82.8% of 1 mM copper(II) and the registered sorption capacity was 0.066 mmol·g^−1^ after 96 h in the kinetic studies [74]. It was even successfully applied in an effluent biotreatment method for the removal of copper(II) from copper-loaded effluents [75]. Although it was suggested that the metal–siderophore interaction may have also contributed to the binding of copper(II) or some other heavy metals via the *P. veronii* 2E strain [76], copper(II) did not promote siderophore genesis. Thus, the interaction of copper(II) in biofilm matrix and complexation by EPSs should be considered the primary detoxification mechanism in *P. veronii* 2E, since the production of soluble ExPs in the presence of divalent cationic heavy metals was enhanced. However, the produced siderophores, which belong to the family of pyoverdines, can be used as an efficient extracting agent for bacteria since they are effective in solubilizing and mobilizing numerous metals with variable affinities [77].

The ATR-FTIR analysis of the *P. veronii* 2E strain’s soluble ExPs displayed the presence of N-acetylaminosugars. The multivariate analysis indicated that the amino groups in these aminosugars were primarily involved in the complexing of copper(II) and zinc(II). However, the carboxylates of polysaccharides in the EPS of *P. veronii* 2E chelated cadmium(II) [78]. The purified ExP fraction of this autochthonous bacterium [73], isolated from sediments associated with the Reconquista River Basin (Buenos Aires Metropolitan Area), was fractionated by anion exchange chromatography resulting in three acidic and one neutral polysaccharide fractions. While these included glucose as the main sugar in all fractions, other components were listed as mannose, glucosamine, fucose, and glucuronic acid [79]. The ATR-FTIR analysis revealed the presence of the amino and carboxylic groups that are most likely responsible for metal complexation in the most abundant ExP fraction (65%), which is α-1-4-glucan, highly substituted with N-acetylglucosamine units.

The composition of the heteropolysaccharide component of *P. stutzeri* AS22, designated as EPS22, has been studied by Maalej et al. [80], who noted that it was mainly composed of mannose, glucose, and carboxyethyl-substituted rhamnose in an approximate ratio of 1.1:1:0.7. It was also suggested that it has enormous potential for biotechnological application due to its advantageous properties over synthetic polysaccharides. Thus, in his later research, Maalej et al. [81] studied the optimal conditions for ExP production by submerged cultures of *P. stutzeri* AS22. The optimal culture conditions were determined to be 30 °C, 250 rpm, 10% inoculum size, initial pH of 8.0, and 24 h of incubation time. The extracted crude ExP’s performance in heavy metal removal was exceptionally good, and it has shown high selectivity for lead(II) (460 mg·g^−1^ ExPs) in a mixed metal solution consisting of 0.1 mmol·L^−1^ lead(II), cobalt(II), iron(II), copper(II), and cadmium(II). Interestingly, the zinc(II) and cadmium(II) uptakes in one-metal sorption experiments were negligible, reaching up to 8 mg·g^−1^ ExP sorption capacity.

Meena et al. [82] tested ten bacterial isolates collected at the rhizosphere of a leguminous plant. Only the *P. stutzeri* TN_AlgSyn showed the capability of synthesizing ExPs whose major component was uronic acid (83%). Further analysis using NMR and FTIR spectroscopy revealed guluronic and mannuronic acid residues, which are the two building blocks of alginate. The synthesized bacterial alginate was then successfully tested for the removal of chromium(VI), lead(II), and cobalt(II) from aqueous media with an adsorption rate over 96%.

Thorgersen et al. [83] noted that exopolysaccharide production via *P. stutzeri* RCH2 is linked to uranium(VI) resistance under anaerobic conditions, since the expression of genes involved in the formation of exopolysaccharides is induced upon strain exposure to U(VI)O_2_^2+^. Marqués et al. [84] recovered ExPs from the culture of *Pseudomonas* sp. EPS-5028 by precipitation with ethanol. The following batch sorption experiment showed that the sorption efficiency of 0.1% (*w*/*v*) polysaccharide was independent of temperature but highly pH-sensitive, since the uptake declined significantly below pH 5. The maximum uranium(IV) removal was 96 µg·mg^−1^. More importantly, the desorption with 0.1 M sodium carbonate released up to 98% of metal bound to ExPs, indicating that uranium(IV) is coupled with the ligands that are easily substituted with carbonate. However, when living cells are involved, uranium is accumulated intracellularly as needle-like fibrils [85]. Nevertheless, this strain belongs to procaryotes, which have shown unique efficiency in trapping uranium, alongside the species of *Bacillus mucilaginosus* ACCC 10012, *Pseudomonas* sp. MGF-48, *Streptomyces* sp., and *Bacillus licheniformis* ATCC 14580 [86].

The FTIR analysis of EPS and the cell wall of *P. putida* that was exposed to the 10 mg·L^−1^ of cadmium(II) revealed that it is directly complexed with phosphoryl and polysaccharide groups [87]. Therefore, the presence of ExPs increased the viability of the cell. Similar outcomes have been presented by Wei et al. [88], who concluded that the *P. putida* strain’s carboxyl and phosphate groups in EPS (comprising 128 mg·g^−1^ polysaccharides and 290 mg·g^−1^ proteins) increased the total binding sites concentrations for cadmium(II). Unfortunately, the EPS-free cells’ maximum sorption capacity derived from Langmuir isotherm decreased by only 9.7% in comparison to the untreated cells. Furthermore, Ueshima et al. [87] highlighted that there was no significant difference on a per mass basis in the extent of cadmium(II) binding between the components of the EPS and cell wall.

The extended X-ray absorption fine-structure (EXAFS) spectroscopy and equilibrium titration studies performed by Guiné et al. [89] indicated that zinc(II) is primarily complexed with phosphoester, carboxyl, and sulfhydryl ligands in *P. putida* ATCC 12633 biomass and, more importantly, EPSs play the dominant role in zinc retention compared with other cell components (e.g., periplasmic space, outer membrane) with reactive site densities of 16 zinc·nm^−2^. Additionally, Lin et al. [90] noted that the K-edge X-ray absorption near-edge structure (XANES) analysis showed that, besides the cell walls’ and cell membranes’ phospholipids and intracellular thiol-rich proteins, the acidic polysaccharide alginate in EPS played a crucial role in copper(II) binding in *P. putida* CZ1. The authors suggested that the carboxyl functional groups of alginate played a key role in the adsorption process since they predominantly complexed with copper in EPS. Furthermore, reduced copper(I) species were identified in the biofilm microenvironment. Andreazza et al. [91] reported that the *Pseudomonas* sp. strain NA was capable of reducing 23.4 mg·L^−1^ of initial 100 mg·L^−1^ copper(II) after 24 h incubation. This can be linked to the toxicity response of the biofilm to copper(II) exposure [92].

Upadhyay and Srivastava [93] highlighted the role of ExPs in zinc(II) accumulation via the plant-growth-promoting Gram-negative bacterium *P. fluorescens* Psd [94]. Zinc was capable of promoting the ExP synthesis by the bacterium and the exposure to 5 mM zinc(II) enhanced the ExP levels by six times. The PsD strain could immobilize up to 265 mg·g^−1^ (approximately 70%) of zinc(II). Such a positive effect on ExP synthesis can be used in the mitigation of drought or salinity stress in plants to increase agricultural productivity [95]. The biotechnological potential of the genus *Pseudomonas* for heavy metal removal was also highlighted by Vélez et al. [96], who employed *P. nitroreducens*, *P. alcaligenes*, and *P. aeruginosa* for lead(II) sorption.

### 3.2. Wastewater Treatment via Bioflocculation

One of the most common processes in biological wastewater treatment includes the usage of activated sludge for organic or inorganic contaminant removal and decreasing turbidity of biological or non-biological origin. Therefore, the EPS production of the sludge bacteria plays a crucial role in floc formation. Therefore, ExPs of *Pseudomonas* bacilli isolated from the activated sludge have been widely studied for their role in flocculating properties.

The strain *P. aeruginosa* IASST201 isolated from the activated oil sludge could remove up to 68% straight chain hydrocarbon through the entrapment mechanism via bioflocs during the treatment of oil field formation water [97]. Furthermore, the crude bioflocculant, consisting of 62% carbohydrates, showed exceptional 80%, 83%, and 90% removal capacity for nickel, iron, and chromium, respectively. Subramanian et al. [98] noted that the ExP fraction in EPS played the leading role in sludge flocculation via the *Pseudomonas* strain BS2. Similarly, *Pseudomonas* sp. strain 38 A, isolated from a wastewater treatment plant, was used to produce bioflocculant and subsequently the bioflocculation of kaolin clay suspension [99]. The bioflocculant consisted of carbohydrates with little presence of protein and, at optimal conditions, it reached the flocculation activity of 99.9%. Liu et al. [100] successfully tested the reduction in artificial turbidity using the EPS of *P. veronii* L918, whose composition was mainly polysaccharide (77%), and the turbidity reduction reached 93%.

The superiority of the bioflocculant application in contrast to the standard usage of inorganic flocculants (e.g., alum) has been noted by Buthelezi et al. [101], who highlighted the satisfactory performance of various Gram-negative and Gram-positive bacterial bioflocculants, including biopolymers produced by *P. pseudoalcaligenes* and *P. plecoglossicida*. On the other hand, the presence of ExPs may enhance the flocculation efficiency of inorganic-based flocculants. Such is the case of a composite based on the EPS of *P. aeruginosa* ZJU, whose outstanding performance in the flocculation of harmful algal blooms was reported by Sun et al. [102].

The performance of ExPs, however, is highly affected by the synthesis medium composition since it influences the content of biopolymeric substances in bacterial bioflocculant. However, the outcomes can be confusing. Drakou et al. [103] noted that while the ExPs produced by the *P. aeruginosa* strain LVD-10 cultivated on crude glycerol reached flocculation efficiency higher than 80%, and the performance of ExPs collected from the bilge wastewater was inferior, the emulsification properties of the isolated exopolysaccharide matrix were significantly higher in the strain cultivated on bilge wastewaters.

Another critical issue in treating wastewater and effluents is the removal of reactive (azo)dyes. Decolorization usually utilizes both the sorptive properties of ExPs and the degradative capabilities of bacterial consortia dwelling in the EPS. Mao et al. [104] studied the optimal conditions to produce a biopolymer via *P. fluorescens* in brewery wastewater and its use for the removal of reactive light-yellow K-4G and reactive turquoise-blue KN-G. The single-strain performance (*P. alcaligenes* PS-25) of the removal of these dyes has also been studied by Wang et al. [105]. The bioflocculant, comprising primarily of saccharides, reached a satisfactory decolorization of over 90% in both cases.

The usage of bacterial consortia may be less efficient in dye removal, but they can be applied in a wide range of environmental conditions. The consortia of *Klebsiella* sp. PCH427, *Enterobacter* sp. PCH428, and *Pseudomonas* sp. PCH429 degraded only 77% of the synthetic dye mixture [106]. However, the performance was relatively consistent at a wide range of pHs, at high salt concentrations, and low nutrient availability as well.

### 3.3. Soil Reclamation and Remediation

The contamination of soil and the degradation of its physicochemical properties are issues that are the top priorities of remediation strategies worldwide. Thus, the usage of purified bacterial ExPs or the direct in situ application of ExP-producing strains is extensively studied. This is due to their excellent sorptive properties, ability to enhance phytoremediation performance, and capacity to stabilize the soil environment. In the case of the latter, Yi et al. [107] used the ExPs of *Pseudomonas* sp. as a biocement to increase the soil stability and decrease the water permeability of sandy soils. The inoculation by bacteria changed the penetration stress characteristics of subjected soil samples since the ExPs attached to the sand soil particles and, thus, filled, patched, and closed the pores between them. However, when the bacilli are introduced directly, it should be taken into consideration that the production and quality of ExPs alter through time in response to environmental stressors such as drought, salinity, and temperature. Sandhya and Ali [108] reported that the production of ExPs by the *P. putida* GAP-P45 strain was high under drought stress, and increased with increasing stress levels (e.g., up to 1.4 M salinity and temperature of 50 °C). Additionally, the soil aggregation stability increased under stress conditions.

Interesting research about the usage of ExPs in soils for biostimulation was conducted by Fatima and Arora [109]. They studied the plant growth-promoting traits of ExPs isolated from *P. entomophila* PE3, and their effect on salinity tolerance and growth, oil production, and the biochemical aspects of the common sunflower (*Helianthus annuus*) under salinity stress. The field experiments with isolated ExPs introduced to the soil showed significant improvement in the salinity tolerance of the plant and promoted the growth attributes of *H. annuus* in saline fields. Similarly, Tewari and Arora [110] concluded that the ExPs of *P. aeruginosa* PF23 have an important role in stress amelioration under saline conditions, serve as plant growth promoters, and participate in biological control against pathogens.

## 4. Conclusions

In microorganisms, extracellular polymeric substances have a strong protective role against various environmental stressors, e.g., they allow the efficient sequestration of contaminants and potentially harmful substances. Since ExPs are a major component of biofilm comprising extracellular polymeric substances, their immobilizing capabilities and potential applications in environmental protection and the remediation of contaminated sites have been studied extensively. ExPs are also a key constituent of extracellular matrix material secreted by *Pseudomonas* species; thus, we have reviewed the prospects of this ubiquitous genus in the remediation of matrices contaminated with metals and metalloids via sorption and bioflocculation. Here, we have highlighted that ExPs have enormous potential in remediation technologies, including wastewater treatment and soil reclamation. Still, further work will be needed for revealing their full potential in these processes and the successful transfer of experimental outcomes to other industrial branches, such as agriculture and biotechnology.

## Data Availability

Not applicable.
